# Differences in Ratio of Carbon Stable Isotopes among Barley Grain Milling Fractions with Various Concentrations of Beta-Glucans

**DOI:** 10.3390/molecules28155738

**Published:** 2023-07-29

**Authors:** Tom Levanič, Blaž Cigić, Mateja Germ, Ivana Polišenská, Kateřina Vaculová, Igor Pravst, Darja Kocjan Ačko, Ivan Kreft

**Affiliations:** 1 Slovenian Forestry Institute, Večna pot 2, SI-1000 Ljubljana, Slovenia; tom.levanic@gozdis.si; 2 Biotechnical Faculty, University of Ljubljana, Jamnikarjeva 101, SI-1000 Ljubljana, Slovenia; blaz.cigic@bf.uni-lj.si (B.C.); mateja.germ@bf.uni-lj.si (M.G.); darja.kocjan.acko@bf.uni-lj.si (D.K.A.); 3 Agrotest Fyto, Ltd., Havlíčkova 2787, 767 01 Kroměříž, Czech Republic; polisenska@vukrom.cz (I.P.); vaculova@vukrom.cz (K.V.); 4 Nutrition Institute, Koprska ulica 98, SI-1000 Ljubljana, Slovenia; igor.pravst@nutris.org

**Keywords:** beta-glucans, starch, barley, stable isotopes, nutrition, milling

## Abstract

The grains of three barley varieties were milled and sieved to obtain respective milling fractions with a content of beta-glucans (b-G) from 1.4 to 10.7%. The enriched fraction obtained by the extraction and precipitation contained 24.7% of b-G. The differences between the ratio of stable C carbon isotopes were established. Milling fractions with coarse particles had more beta-glucans and a more negative ratio of δ^13^C isotope in comparison to the respective intact barley grain. However, the enriched fraction had a less negative isotope ratio. So, it is not expected that the deviation from the stable isotope ratio of grain in milling fractions is the result of the content of b-G, but it depends on other barley grain constituents. In different parts of barley grain, there are substances with different stable isotope ratios, and by milling and sieving, they are assorted to the same milling fraction with most of the b-G. The method for determining the ratio of a stable carbon isotope in diverse barley grain fractions, applied in this investigation, is potentially opening the possibility for an additional method of screening the concentration of bioactive constituents in barley grain.

## 1. Introduction

Barley (*Hordeum vulgare* L.) is a cereal originating in the Middle East and is used as a staple food. In many countries, it is one of the most important cereal grains. Barley grains could be tightly covered by a husk or naked [1]. Hulled grains are used for food peeled to remove the cover. The covered grains after peeling, or naked grains, could be pearled to remove the aleurone layer and some of the other outer cells of the endosperm. The remaining grain after peeling is barley groat. Barley grains can contain notable amounts of dietary fiber, including beta-glucans (b-G). In the present time, in a large part of the world, barley yield is also used as animal fodder or for the production of beer or spirits [1].

In the production of alcoholic beverages, b-G are a disturbing constituent as it causes a high viscosity of suspensions. To meet the demands of beer production industries, barley breeding is often oriented towards the selection of cultivars with a lower fiber content, including a lower concentration of b-G in the grain. But this breeding orientation is not suited to human nutritional needs in developed countries, where insufficient dietary fiber intake was reported [2,3]. From this point of view, interest is reappearing in barley varieties with a high content of dietary fiber, including a high concentration of b-G. Barley could be a rich nutritional source of soluble fiber, including 1,3/1,4 beta-glucans [4,5,6]. Beta-glucans increase the viscosity of digesta in the intestine, lowering the reuptake of cholesterol and reducing the postprandial concentration of glucose in the blood. The regular consumption of barley contributes to improving the values of serum TC and LDL cholesterol and preventing cardiovascular diseases [7,8,9]. A disease risk health claim was also authorized in the European Union, enabling the marketing of foods (that contain at least 1 g of b-G per quantified portion) with a claim that barley beta-glucans have been shown to lower blood cholesterol, which is a risk factor in the development of coronary heart disease [10]. Possibilities for increasing the concentration of b-G during the milling process are therefore of special interest [11]. The impact of b-G on the viscosity of ingested cereal material is limited by the presence of the enzymes beta-glucanases, which are in wet conditions, reducing the molecular weight of b-G. However, the heating of barley feed materials may reduce the activity of beta-glucanase [12]. Barley containing a higher b-G concentration also causes a higher viscosity in poultry feed. The addition of beta-glucanase to the poultry feed can resolve this issue [13].

A considerable part of b-G in barley is located in the endosperm cell walls. Thick cell walls may protect cells from water and enzymes entering. So, the content of cells is protected from digestion (cage effect). The digesta from varieties with high levels of b-G are thicker than from the varieties with low levels of b-G [14].

In barley grain cells, b-G are located in combination with proteins as a wrap on the surface of the starch granules. Barley grain b-G can restrict the availability of water for the enzymatic reactions of starch in the granules and inhibit the process of glucose release from starch granules during gastric digestion [15]. Barley b-G, during its fermentation by gut bacteria, contributes to the appearance of short-chain fatty acids. As a result, postprandial blood glucose levels rise slower, and insulin secretion is reduced [8].

In the last decade, there has been a rise in the awareness of the importance of b-G in barley breeding for obtaining cultivars with higher b-G content for food products [16,17]. A high b-G mutant lys5.f, from a set of barley lys genes, leads to a significant increase in the production of mixed-linkage b-G molecules [18]. Metabolic patterns, including carbohydrate metabolism, were studied for the barley mutants, whereas growth temperature and drought can primarily affect metabolic pathways [18,19,20,21].

This investigation aims to find the differences in stable isotope constitutions and ratios in the barley milling fractions with the diverse concentration of b-G, thus suggesting the possibilities of using stable isotopes as markers and additional tools in tracing the pathways of metabolite synthesis in barley, regarding the link of genetic and environmental factors with the seed phenotype. This study aimed to elucidate the possibility of tracking the conditions of b-G synthesis by assessing the differences in the ratio of C stable isotopes. 

## 2. Results and Discussion

**Figure 1:**
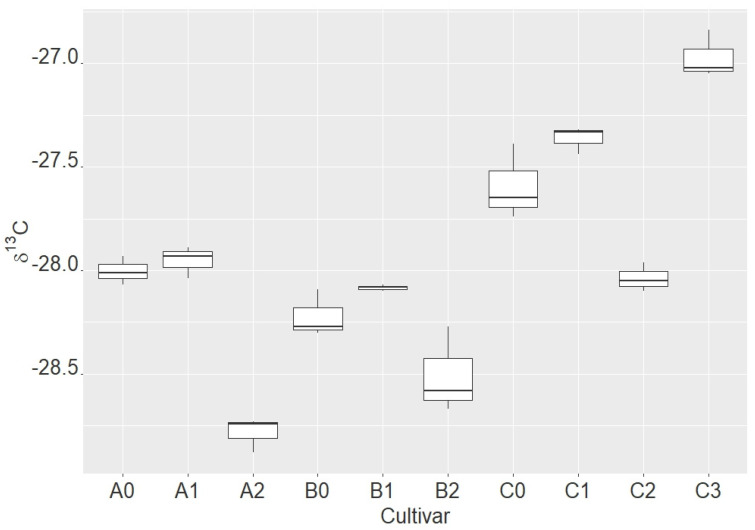
Stable carbon isotope ratio (δ^13^C) in milling fractions of each of three cultivars of barley (A (0–2) = Sandra, B (0–2) = Hyvido, C (0–3) = AF Cesar).

The results of the stable carbon isotope ratio (δ^13^C) in the milling fractions of the three barley varieties studied are shown in Figure 1 and Table S1. The differences, based on Tukey’s HSD, in stable carbon isotope ratios among the milling fractions are shown in Table 1. We confirmed the differences in stable carbon isotope ratios among the barley cultivars as well as among the milling fractions within each cultivar. The only exception is the first milling fraction of cv. Sandra, which does not differ from the value for the whole seed. The first milling fraction of cv. Hyvido is not different from the total seed value, but both fractions have a different ratio compared to each other. Similarly, the second fraction of cv. Sandra is not significantly different in ratio from the second fraction of the Hyvido variety. The second fraction of AF Cesar does not differ in the ratio of stable carbon isotopes from the first milling fraction of cv. Sandra or from the first milling fraction of cv. Hyvido.

It is interesting to note that the second milling fractions of the barley samples differ in δ^13^C (stable carbon isotope ratio) from the respective whole barley samples of the three varieties studied, and they have a much lower negative carbon isotope ratio compared to their whole grains. In contrast, fraction C3 has the least negative stable carbon isotope values compared to all the samples studied (Table 1). 

**Figure 2:**
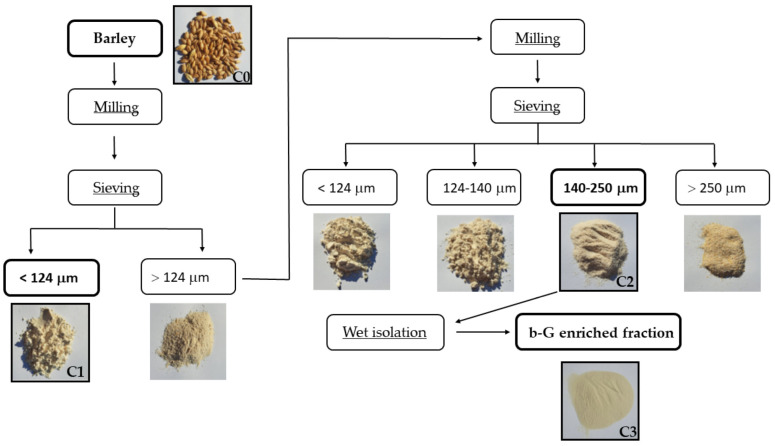
Flowchart of milling, sieving, and preparation of the b-G-enriched fraction by wet isolation from barley cultivar AF Cesar. Barley (C0), the fraction with the lowest beta-glucan content (C1), the fraction with the highest beta-glucan content obtained by milling and sieving (C2), and the b-G-enriched fraction (C3) obtained by extraction and precipitation from C2 were used for stable carbon isotope analysis.

The grains of the three barley varieties were separated by mechanical milling and sieving into two samples each with different particle sizes (Figure 2, Table 2). It is expected that at least some of the particles in the two milling fractions originated from different parts of the grain. The differences in the stable carbon isotope ratio in the respective fractions indicate that the origin of the materials in the milling fractions was different. 

In all three barley varieties, the crude milling fraction, with a higher concentration of b-G, had lower negative values of stable carbon isotopes ratio than the whole grain or the fine milling fraction. On the contrary, b-G-enriched fraction C3 had a significantly higher negative ratio of stable carbon isotopes than any other milling fraction studied. It could not be expected that mainly b-G themselves are the source of the differences in stable carbon isotope ratios, but it is probably due to other substances entering into the same milling fraction as b-G [4,5,22,23] or the absence of some substance, for example, the proteins which were removed during the enrichment of b-G in this fraction. Additionally, in three studied barley varieties, there were some significant differences in the stable carbon isotope ratios between the fractions.

The milling fractions of barley grain are not just mixtures of compounds. They are organized in the cellular structures, including cell wall structures with b-G, and starch grains enveloped in the proteinaceous/carbohydrate matrix, as the remainder of cytoplasm after the drying of the grain [22,23,24,25,26,27,28]. So, the δ^13^C of the seed fraction is a result of all contents of the sample, not necessarily a predominance of the b-G.

Hydrothermal pre-treatment can change the properties of a plant material and thus alter its effect on the organism [22,23,24,25,26,27,28]. Plant material is subjected to microbial activities during the digestion in the gut, which may change the physical (viscosity) and chemical properties of ingested materials [22,29,30,31,32,33,34]. 

The stable carbon isotope ratios in barley grain are a part of its phenotype. The difference in stable carbon isotope ratio in different parts of barley grain and its milling fractions offers a possibility to track the phenotype back to the interaction of genetic and ecological conditions during the growth and development of the constituents of barley grain, including germ and endosperm. 

Hussain et al. [32] evaluated the carbon isotope ratio in barley in connection with salinity tolerance potential and established differences among cultivars. In the study of milling fractions in wheat, a similar range of the ratio of δ^13^C was obtained in wheat in comparison to barley [33]. The stable carbon isotope ratio values in some milling fractions of wheat were different than in wholemeal flour. In contrast, stable nitrogen isotopes were more stable, and there was no significant difference between milling fractions and whole wheat grains [31,32,33,34].

Carbon isotope ratio data should be able to support research linking genetic, environmental, and agrotechnical factors to the resulting seed phenotype. In barley, this would allow the development of unique, agro-economically important models for optimal dietary fiber production and utilization as food or feed.

## 3. Materials and Methods

### 3.1. Materials

Grain of three cultivars of barley (*Hordeum vulgare* L.), were used: hulled winter cv. Sandra, hulled spring cv. Hyvido, and naked spring cv. AF Cesar. The sample of cv. AF Cesar was cultivated at the Agricultural Research Institute (Agrotest Fyto, Ltd., Kroměřiž, Czech Republic) in 2018, and growing conditions were described by Cajzek et al. [35]. Cv. Sandra and cv. Hyvido were obtained in the same year from the Biotechnical Faculty, University of Ljubljana.

We analyzed milling fractions of three barley cultivars: Sandra Agrosaat with a low concentration of b-G in grain (3.6% in sample), Hyvido with a medium b-G concentration (4.0% in sample), and AF Cesar with high b-G concentration in grain (5.7% in sample) (Table 2).

After the harvest, barley seed samples were stored in paper bags at 25 °C in dark space until milling. Samples with 13.2% moisture were milled using a stone mill set (Osttiroler; TYP A130). After milling, the moisture content was 12%. Milling conditions were described by Cajzek et al. [35] (2020). Flour fractions with differences in particle size were obtained with the Osttiroler sifting machine using the combination of sieves with aperture openings of 124, 140, and 250 µm.

For each flour fraction, beta-glucan content was determined in three independent parallels (weighing), and the average content was calculated. A non-parametric Mann–Whitney test based on the data ranking was used for the statistical analysis. Differences in the content of beta-glucans in all fractions of the same cultivar were significant at the *p* < 0.05 level. The research was conducted for one year, and this is a preliminary study.

### 3.2. Analysis of b-G

Barley samples were homogenized using the metal ball homogenizer (Retsch MM 400) and analyzed with the mixed-linkage b-G assay kit (Mixed-linkage β-glucan Assay Kit, Megazyme Intl., Bray, Ireland) as described by Cajzek et al. [35].

### 3.3. Preparation of b-G-Enriched Fraction C3

Amount of 40 g of fraction C2 was suspended in 800 mL of boiling miliQ water. After 10 min of boiling, the suspension was cooled to 25 °C and centrifuged for 10 min at 4000× *g* (Rotanta 460R, Hettich, Tuttlingen, Germany). The supernatant was deproteinized by 1.2 U/L of Alcalase^®^ 2.4 L (Merck, Darmstadt, Germany) at 60 °C for 3 h, followed by centrifugation for 10 min at 4000× *g*. The b-G in the supernatant were precipitated by adding ethanol to 40% (*v*/*v*). The suspension was stored for 12 h at 4 °C, and precipitate rich in b-G was lyophilized to dryness (fraction C3).

### 3.4. Stable Carbon Isotope Analysis

Samples of barley milling fractions were weighed (0.687–0.732 mg) into tin capsules and combusted. Combustion was conducted at 950 °C in the presence of pure oxygen using a ELEMENTAR Vario PYRO Cube elemental analyzer (Elementar, Langenselbold, Germany) interfaced to the IsoPrime100 stable isotope ratio mass spectrometer ELEMENTAR ISOPRIME 100 (Isoprime, Elementar, Langenselbold, Germany) in continuous flow mode. Each sample was run in triplicate.

Certified reference materials (USGS40 and USGS41 for carbon) and in-house laboratory standards (spruce cellulose, acetanilide, and corn flour) were used to control the accuracy and precision of the measurements. The analytical precision was <0.1‰ for carbon (expressed as the standard deviation of repeated measurements of in-house working standards (n = 30)). Stable isotope results (δ^13^C) are reported in terms of relative delta (δ) notation value as a quotient between ^13^C/^12^C ratio of the sample and international reference material (Vienna PeeDee Belemnite (VPDB)), expressed in parts per thousand notation (‰)—see Equation (1):

(1) $$\delta^{13}C\left(‰\right)=\left(\frac{{}^{13}C/{{}^{12}C}_{sample}}{{}^{13}C/{{}^{12}C}_{standard}}\right)\times 1000$$

where ^13^C/^12^C_standard_ is the Vienna PeeDee Belemnite (VPDB) standardized coefficient [36] and ^13^C/^12^C_sample_ is the measured value [36,37]. All values of stable isotope ratios are expressed in per-mill notation (‰).

## 4. Conclusions

There are different contents of b-G and differences in the stable carbon isotope ratio in barley grain fractions obtained by milling and sieving. Study results indicate that in respective milling fractions, there are b-G, starch, proteins, lipids, and secondary metabolites, which result from different enough metabolic pathways. Due to the involvement of carbon substances in different metabolic procedures, the heavier carbon isotope (^13^C) has more difficulties entering metabolic reactions, as their involvement needs more energy. So, this is an advantage for a lighter carbon isotope (^12^C).

The main substance for such differentiation of stable carbon isotopes is not necessarily b-G. Therefore, it is not likely that b-G themselves are the source of deviation of b-G-rich fraction from the stable isotope ratio of grain and fine milling fraction. Additionally, different parts of barley grain result during milling in fractions with different stable isotope ratios. Some grain metabolites result from milling and sieving assorted in the same milling fraction with b-G. This means that they must be allocated to the same grain structures as b-G. Further studies on more detailed separation of milling fractions are needed to establish which barley grain substances contribute to the differences in stable carbon isotope ratio in different parts of barley grain. When this is established, it may open the possibility for the development of an additional method for screening the concentration of substances in barley grain.

## Figures and Tables

**Table 1:** Comparison of differences in barley samples based on the Tukey’s HSD with regard to the stable carbon isotope ratio (levels of significance: *—5%, **—1%, ***—0.1% or higher, ns—not significant).

	A1	A2	B0	B1	B2	C0	C1	C2	C3
A0	ns	***	ns	ns	***	***	***	ns	***
A1	X	***	ns	ns	***	*	***	ns	***
A2		X	***	***	ns	***	***	***	***
B0			X	ns	ns	***	***	ns	***
B1				X	**	***	***	ns	***
B2					X	***	***	**	***
C0						X	ns	**	***
C1							X	***	*
C2								X	***
C3									X

**Table 2:** Percentage of beta-glucans (b-G) in barley samples (with 12% moisture).

Barley Samples	Milling Fractions	Concentration of b-G [%]
Sandra (A0)	Whole barley	3.6
Sandra (A1)	Fraction ˂ 124 µm	1.5
Sandra (A2)	Fraction 140–250 µm	5.8
Hyvido (B0)	Whole barley	4.0
Hyvido (B1)	Fraction ˂ 124 µm	1.8
Hyvido (B2)	Fraction 140–250 µm	7.2
AF Cesar (C0)	Whole barley	5.7
AF Cesar (C1)	Fraction ˂ 124 µm	1.4
AF Cesar (C2)	Fraction 140–250 µm	10.7
AF Cesar (C3)	b-G-enriched fraction	24.7

## Data Availability

The data presented in this study are available in the article.

## Supplementary Materials

The following supporting information can be downloaded at: https://www.mdpi.com/article/10.3390/molecules28155738/s1, Table S1: Stable carbon isotope ratio with mean, standard deviation, median and minimum and maximum values (all values are displayed in per mill notation ‰).

## Sample Availability

Samples of the compounds are not available from the authors.

## Abbreviation

b-G	beta-glucans
