# Supplementary material for: Differences in Ratio of Carbon Stable Isotopes among Barley Grain Milling Fractions with Various Concentrations of Beta-Glucans
Source: Molecules. 2023 Jul 29;28(15):5738. doi: 10.3390/molecules28155738 (PMC10420649; doi:10.3390/molecules28155738)
Supplement: Supplementary file 1 [file molecules-28-05738-s001.zip › molecules-2452549-supplementary.pdf]

Table S1. Stable carbon isotope ratio with mean, standard deviation, median and minimum and maximum values (all values are displayed in per mill notation ‰).

Cultivar	Milling fraction	mean	sd	median	min	max
Sandra	A0	−28.00	±0.07	−28.01	−28.07	−27.93
	A1	−27.95	±0.08	−27.93	−28.04	−27.89
	A2	−28.78	±0.08	−28.74	−28.88	−28.73
Hyvido	B0	−28.22	±0.11	−28.27	−28.30	−28.09
	B1	−28.08	±0.02	−28.08	−28.10	−28.07
	B2	−28.51	±0.21	−28.58	−28.67	−28.27
AF Cesar	C0	−27.59	±0.18	−27.65	−27.74	−27.39
	C1	−27.36	±0.07	−27.33	−27.44	−27.32
	C2	−28.04	±0.07	−28.05	−28.10	−27.96
	C3	−26.97	±0.11	−27.02	−27.05	−26.84
